# The environment, the tick, and the pathogen – It is an ensemble

**DOI:** 10.3389/fcimb.2022.1049646

**Published:** 2022-11-02

**Authors:** Jannelle Couret, Samantha Schofield, Sukanya Narasimhan

**Affiliations:** ^1^ Department of Biological Sciences, College of Environment and Life Sciences, University of Rhode Island, Kingston, RI, United States; ^2^ Section of Infectious Diseases, Department of Internal Medicine, Yale University School of Medicine, New Haven, CT, United States

**Keywords:** *Ixodes scapularis*, tick, *Borrelia burgdorferi*, environmental factors, microbiome

## Abstract

*Ixodes scapularis* is one of the predominant vectors of *Borrelia burgdorferi*, the agent of Lyme disease in the USA. The geographic distribution of *I. scapularis*, endemic to the northeastern and northcentral USA, is expanding as far south as Georgia and Texas, and northwards into Canada and poses an impending public health problem. The prevalence and spread of tick-borne diseases are influenced by the interplay of multiple factors including microbiological, ecological, and environmental. Molecular studies have focused on interactions between the tick-host and pathogen/s that determine the success of pathogen acquisition by the tick and transmission to the mammalian host. In this review we draw attention to additional critical environmental factors that impact tick biology and tick-pathogen interactions. With a focus on *B. burgdorferi* we highlight the interplay of abiotic factors such as temperature and humidity as well as biotic factors such as environmental microbiota that ticks are exposed to during their on- and off-host phases on tick, and infection prevalence. A molecular understanding of this ensemble of interactions will be essential to gain new insights into the biology of tick-pathogen interactions and to develop new approaches to control ticks and tick transmission of *B. burgdorferi*, the agent of Lyme disease.

## Introduction

*Ixodes scapularis* is one of the predominant tick vectors of *B. burgdorferi*, the agent of Lyme disease, and the most common tick-borne disease in North America emerging from the northeastern and north central United States foci, where 95% of Lyme Disease (LD) cases occur (Fleshman et al., 2021). Movement of *B. burgdorferi* within tick tissues, its maintenance across life stages as ticks molt, as well as its proliferation and transmission to vertebrate hosts during tick bloodmeal engorgement may all be subject to environmental influence, be it biotic factors such as the composition of the microbiome, abiotic factors such as temperature and humidity, and the responses of the tick to the spirochete, the vertebrate host and to the environmental components. There is an increasing understanding that environmental conditions affect tick physiology, population dynamics, and *B. burgdorferi* acquisition and transmission (Dumic and Severnini, 2018). This may be mediated through different components of tick physiology, immune processes, or host activity. Modeling studies have shown that environmental effects on *I. scapularis* can influence phenology and *B. burgdorferi* dynamics across geographic regions (Perret et al., 2000; Ogden et al., 2005; Ogden et al., 2008; Gaff et al., 2020). Further, the population dynamics of ticks is shaped by host species availability and behavior (Lord, 1992; Tosato et al., 2021). To understand the collective impacts of biotic and abiotic factors on tick biology and on its vectorial capacity is the holy grail of tick research.

## Abiotic factors and tick-pathogen interactions

Studies on the direct impacts of abiotic climatic factors on *I. scapularis* under natural conditions have demonstrated that both temperature and humidity impact tick survival (Lindsay et al., 1995; Bertrand and Wilson, 1996) and host-seeking success (Randolph and Storey, 1999; Randolph, 2004; Ginsberg et al., 2017) and, thus have a direct impact on spirochete infection prevalence in endemic areas. Understanding the direct impacts of these factors on ticks has been foundational to hypothesis about the geographic range, seasonal phenology, and survival of *I. scapularis* (Ogden et al., 2005) as well as the fitness of the human pathogens they transmit (Ogden et al., 2008; Ogden et al., 2014). Much of our current understanding of the role of the environment on *I. scapularis* and *B. burgdorferi* transmission dynamics comes from evaluation of effects on tick populations by estimating population measures such as the timing of peak abundance of each life stage (Ogden et al., 2018) or peak host seeking (Ginsberg et al., 2017). Such measures, while informative, are phenomenological such that the geographic variation observed in *I. scapularis* phenology has been associated with abiotic (Ogden et al., 2018) and biotic factors (Tsao et al., 2021). There is a burgeoning interest in expanding our mechanistic understanding of the drivers of *I. scapularis* phenotypic expression using experimental methods to estimate effect measures of light level (Perret et al., 2003), humidity, temperature, and population differences in ticks (Arsnoe et al., 2015) and how these influence the transmission dynamics of *B. burgdorferi*.

### Impact of temperature on tick -*B. burgdorferi* interactions

Temperature is a critical environmental factor, with evidence across diverse insect taxa establishing its importance in driving the development (Rebaudo and Rabhi, 2018), biophysical processes (Gillooly et al., 2001), and population growth (Savage et al., 2004). Fewer works have focused on the effect of temperature in longer lived arthropods, whose life stages span seasons, and years with notable exceptions (Sato and Sato, 2015). The phenological timing of peak abundance in the post-egg life stages of *I. scapularis* vary by region (Ogden et al., 2018) and are at least, in part, likely driven by climatic factors including temperature, relative humidity, and daylight. *I. scapularis* ticks spend most of their life cycle in the environment rather than on vertebrate hosts, and must contend with temperature fluctuations that occur daily, the changes in temperature over months, seasons, and years, and the cumulative effects of temperature over their lifetime. Moreover, their behavioral repertoire includes walking within and questing above the vegetative layer, and little is known regarding the impact of the type of vegetation on the fluctuations in temperature ticks experience as they move within and without of leaf litter. Importantly, arthropod-microbe symbioses, whether mutualistic or pathogenic, can alter the effects of temperature on arthropod hosts and may even mitigate the stress of heat shock experienced by arthropod hosts during a vertebrate blood meal (Neelakanta et al., 2010; Roma et al., 2019; Lemoine et al., 2020).

**Figure 1:**
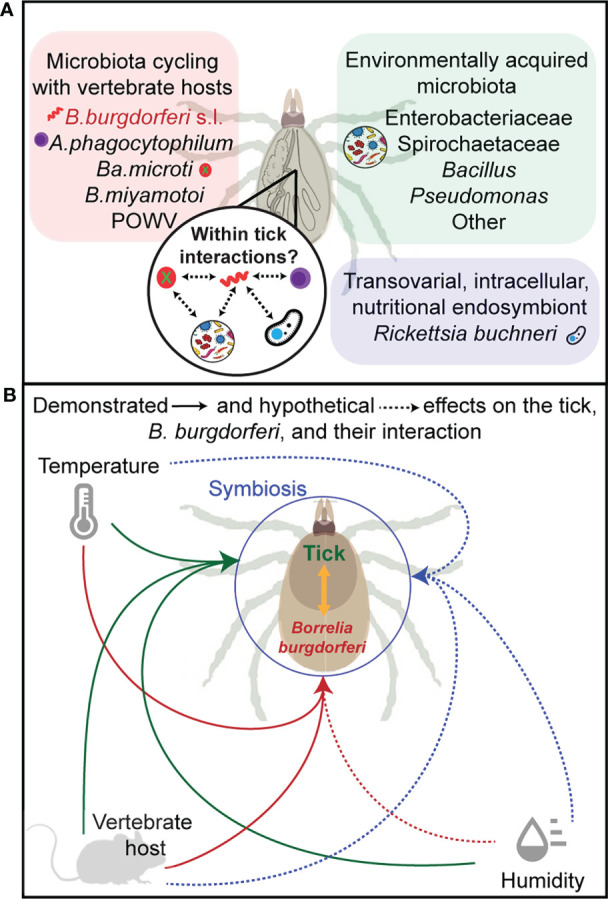
Partitioning the microbiome. Panel **(A)** shows the components of the microbiome in *Ixodes scapularis* as partitioned by route of transmission including environmental acquisition (green), transovarial transmission (blue), and *via* a vertebrate blood-meal (pink) (*B. burgdorferi* s.l, *Borrelia burgdorferi*, sensu lato; *A. phagocytophilum*, *Anaplasma phagocytophilum*; *Ba*. *microti*, *Babesia microti*; *B. miyamotoi*, *Borrelia miyamotoi*; POWV, Powassan virus). Panel **(B)** illustrates the hypothetical (dashed arrows) and demonstrated (solid arrows) effects on gene expression, phenotypic expression, or fitness of the tick (green), *B. burgdorferi* (red), and their symbiotic interactions (blue) for each abiotic factor (temperature and relative humidity), and for vertebrate host factors such as immune response to parasitism.

Temperature has a significant impact on *B. burgdorferi* (Tilly et al., 2008) serving as a critical cue to turn on or off *B. burgdorferi* genes important for colonization of the tick, transmission from the ticks to the vertebrate host and for survival in the host (Carroll et al., 2001; Revel et al., 2002; Ojaimi et al., 2002; Ojaimi et al., 2003). Shifting *B. burgdorferi* from 23 to 37°C, representing ambient temperatures of the tick or the mammalian host, was shown to alter *B. burgdorferi* transcriptome (Ojaimi et al., 2002; Tokarz et al., 2004) and these alterations are critical for *B. burgdorferi* survival in these vastly different milieus (Radolf et al., 2012). Clearly, temperature alone does not fully recapitulate the changes that occur in the vertebrate or invertebrate host (Mulay et al., 2009; Iyer and Schwartz, 2016) but it does highlight the vulnerability of *B. burgdorferi* transcriptome to alterations in temperature. Whether changes in ambient temperature that ticks must face in different regions of the USA would similarly impact *B. burgdorferi* gene expression is not known. For example, would increasing ambient temperatures increase expression of OspC, a critical outer surface protein of *B. burgdorferi* (Pal et al., 2004; Tilly et al., 2006) whose expression is regulated by changes in temperature (Schwan et al., 1995), and enhance *B. burgdorferi* transmission to the murine host? A simple binary interaction could theoretically produce such an outcome. Ginsberg et al. (2021) suggested that higher temperatures in the southern USA may cause desiccation stress, possibly resulting in ticks remaining under the leaf litter and thus impacting host seeking behavior of *I. scapularis.* In addition, the availability of hosts such as lizards that are easily accessible under the leaf litter and that readily clear *B. burgdorferi* infections further likely contributes to the absence of a correlation between tick abundance, infection prevalence, and Lyme disease incidences in the southern USA. These observations underscore the need to bear in mind that changes in ambient temperatures would simultaneously impact tick biology in complex ways and in-turn have a functional consequence on vectorial capacity (Schematically summarized in **Figure 1**).

### Impact of humidity on tick -*B. burgdorferi* interactions

The impact of humidity on tick survival and host-seeking behavior is profound (Ginsberg et al., 2017; Fleshman et al., 2021), and *I. scapularis* may be more sensitive to perturbations in humidity than temperature. Blacklegged ticks spend >95% of their lives off hosts, where larvae and nymphs are particularly subject to intense selection due to sensitivity to desiccation, and therefore are restricted to areas where the relative humidity approaches 100%, i.e., in the leaf litter and low-lying vegetation of the forests they inhabit (Perret et al., 2003; Berger et al., 2014). As nymphal *I. scapularis* frequently climb above the leaf litter to host-seek (Arsnoe et al., 2019), the ticks lose water through their integumentary space as they respire and return to below the leaf litter to rehydrate as needed. In *I. scapularis*, the consequences for the daily drops in humidity (below 85% RH) have cumulative survival consequences (Ginsberg et al., 2017). Importantly, tick nutritional reserve levels have been shown to relate negatively to tick movement and the drive to maintain adequate hydration (Crooks and Randolph, 2006; Ginsberg et al., 2017). This suggests that off-host survival is linked to the quality and quantity of the bloodmeals which, after hatching, are required to progress through the three motile life stages, larva, nymph, and adult.

Water absorption in ticks is dependent on their environment. In humid environments, passive absorption is the primary mechanism (Fielden and Lighton, 1996). Active absorption of water vapor can also occur once a certain threshold is reached (the “critical equilibrium humidity”) leading to additional water uptake. The critical equilibrium humidity for ticks generally lies between 75% to 96% relative humidity (Rodgers et al., 2007). Questing as a host-seeking behavior makes *Ixodes scapularis* “sit and wait” predators. This type of arthropod predators have comparatively low Standard Metabolic Rates (SMR), as demonstrated in spiders (Greenstone and Bennett, 1980). Though spiders are considered to have low SMRs, ticks have an SMR that is 12% of that (Lighton and Fielden, 1995). A low SMR rate allows ticks to maintain respiratory substrate (energy stores) for longer periods of time, and results in less frequent occurrences of the burst phase of their respiratory cycle. Active water absorption is energetically costly, as shown by increased metabolic rates in dehydrated *Dermacentor andersoni* when placed in highly humid environments (Fielden and Lighton, 1996), that was accompanied by net water gain.

It was shown in *Culex pipiens* mosquitos that exposure to dehydration resulted in depletion of fat reserves and this had a significant impact on egg production (Benoit et al., 2010). Little is known regarding the patterns of respiration and hydration of *I. scapularis*, or the impact of the percent relative humidity in the air on tick survival and fecundity in association with *B. burgdorferi.* Multiple studies have confirmed that *I*. *ricinus* has higher rates of survival in unfavorable thermo-hygrometric conditions when infected with *B. burgdorferi* (Benelli, 2020). Body composition analysis of *Ixodes ricinus*, the European Lyme disease vector, showed that *B burgdorferi*-infected *I. ricinus* have a higher fat content than uninfected (Herrmann et al., 2013). In theory, this allows for greater energy reserves, i.e., longer periods that ticks can survive in the environment before finding the next bloodmeal, and reserves for the caloric expenditures of questing above the leaf litter providing a potential mechanistic basis for tick-microbe-environment interactions (**Figure 1**).

## Tick-*B. burgdorferi* interactions

Microbes can mediate arthropod host behavior to increase the probability of transmission, a phenomenon that has been demonstrated in insect-borne vertebrate pathogens such as *Trypanosoma* spp. and Dengue (Hurd, 2003; Lefevre and Thomas, 2008). Behaviors affected by parasites and viruses range from feeding behaviors, to reproduction and locomotion (Ribeiro, 2000; Rogers et al., 2002; Lacroix et al., 2005). Within the cycle of transmission by which it is maintained, *B. burgdorferi* is not pathogenic to ticks. Small vertebrate hosts involved in *B. burgdorferi* enzootic transmission cycle, from which the immature stages of *I. scapularis* (the blacklegged tick) take obligatory blood meals, tolerate *B. burgdorferi* infection. Evidence suggests that the ecological association between *I. scapularis* and *B. burgdorferi* may be characterized as a facultative symbiont. Thus, the language around *B. burgdorferi* as a pathogen, while appropriate in human studies, contradicts the nature of the ecological interactions between *B. burgdorferi* and arthropod hosts in the enzootic cycle. The non-pathogenic status of *B. burgdorferi* for *I. scapularis* and the primary enzootic reservoir host *Peromyscus leucopus* (the white footed mouse) is not in dispute (Schwanz et al., 2011). Yet even in studies focused on within-tick interactions*, B. burgdorferi* is often described as pathogenic. Benelli (2020) reviewed the hypothesis of parasite manipulation in tick-borne pathogens, a theory which suggests that tick traits are adaptively altered to improve pathogen fitness, asserting that, particularly for Lyme disease-causing spirochetes, this is a neglected area of research that warrants investigation.

There is increasing evidence that tick-*B. burgdorferi* association has a beneficial impact on the tick, and in-turn also influences *B. burgdorferi* transmission success (summarized in **Figure 1**). *Ixodes ricinus* ticks infected with *B. burgdorferi* sensu lato survive longer when exposed to unfavorable temperature and humidity conditions (Herrmann and Gern, 2010), have higher fat content (Herrmann et al., 2013; Couret et al., 2017) and increased walking behavior compared to uninfected ticks (Herrmann and Gern, 2015). Our observations on *I. scapularis -B. burgdorferi* also phenocopied these observations. We found greater and more rapid engorgement of larval *I. scapularis* upon successful *B burgdorferi* acquisition (Couret et al., 2017). *B. burgdorferi*-infection has also been shown to influence *I. scapularis* gene expression (Ramamoorthi et al., 2005; Kim et al., 2021; Tang et al., 2021). For example, the increase in expression of Salp15, a tick salivary immunomodulator, was shown to enhance *B. burgdorferi* transmission to the murine host (Ramamoorthi et al., 2005). Due to the reduced genome of *B. burgdorferi*, it has to scavenge nutrients such as carbohydrates, amino acids and lipids from it arthropod or mammalian host (Corona and Schwartz, 2015; Kerstholt et al., 2020; Gwynne et al., 2022). Whether the impact of *B. burgdorferi* on metabolic pathways of the tick (Corona and Schwartz, 2015) and consequent changes in nutritional reserves could have consequences on tick survival, development timing, respiration, locomotion, host-seeking and fecundity remains to be experimentally verified. A molecular and mechanistic understanding of how *B. burgdorferi* increases lipid and energy reserves in the tick or alters tick gene expressions to facilitate transmission and survival in the host will be critical to gain new insights into tick-*B. burgdorferi* interactions. This understanding may be exploited to develop strategies towards tick control measures and to prevent *B. burgdorferi* transmission. For example, key genes/pathways modulated by *B. burgdorferi* and that allow increased lipid reserves may be utilized to develop reservoir host-targeted vaccines to impair tick survival to reduce tick populations. Conceivably, specific gene functions may also be targeted using small molecule inhibitors-potentially developing specific and novel acaricides. Genes that facilitate *B. burgdorferi* transmission may be similarly developed as vaccines targeting reservoir host or for human use.

## Tick- *B. burgdorferi*-pathogenic microbiota interactions

*I. scapularis* can be coinfected *with* other human pathogens including *Anaplasma phagocytophilum, Borrelia miyamotoi, Babesia microti*, and Powassan virus (Stafford et al., 1999; Scoles et al., 2001; Tokarz et al., 2010; Hermance and Thangamani, 2017). The interactions between the tick and these pathogens in the context of biotic and abiotic factors may additionally influence *B. burgdorferi* survival in the tick and its transmission to the vertebrate host (Ginsberg, 2008). Busby et al. (2012) and Villar et al. (2010) showed that *A. phagocytophilum* infection of ticks causes the upregulation of stress response proteins such as heat shock proteins (HSP) and Subolesin, an Ankyrin-like transcriptional regulator of multiple cellular functions (de la Fuente et al., 2008). *A. phagocytophilum*-infected ticks are therefore protected from heat stress and demonstrate increased questing speed (Busby et al., 2012), an adaptation that could increase tick survival by increasing the chances of finding a host to acquire a bloodmeal. *B. burgdorferi* infection also appears to enhance the ability of ticks to survive desiccation (Herrmann and Gern, 2010; Herrmann et al., 2013), facilitating host seeking over longer distances (Herrmann and Gern, 2015). Whether warming climatic conditions would result in increased survival of ticks coinfected with *B. burgdorferi* and *A. phagocytophilum* would require a deeper understanding of interactions between the tick, the vertebrate host, and these pathogens. Neelakanta et al., 2010 showed that *A. phagocytophilum*-infection of *I. scapularis* results in increased expression of an anti-freeze glycoprotein and this increase allows ticks to survive cold temperatures significantly better than uninfected or *B. burgdorferi*-infected ticks. Whether co-infected ticks similarly survive cold stress has not been examined. It is worth noting that while *B. burgdorferi* colonization of the tick gut is enhanced in the presence of an intact peritrophic matrix (Narasimhan et al., 2014), *A. phagocytopilum* infection of the tick is enhanced when the peritrophic matrix integrity is compromised (Abraham et al., 2017). These potentially contrasting interactions between tick-*B. burgdorferi* and tick-*A. phagocytophilum* during pathogen acquisition by the tick may have functional consequences on the prevalence of coinfected ticks in nature. Consistent with this, Levine and Fish (Levin and Fish, 2001) showed that when larval ticks fed on white-footed mice coinfected with *A. phagocytophilum* and *B. burgdorferi* the ability of the larval ticks to efficiently acquire these pathogens is impaired. In contrast, Levine and Fish (Levin and Fish, 2000) showed that coinfection of ticks with *A. phagocytophilum* and *B. burgdorferi* did not impact efficiency of transmission of either pathogen to the murine host. Further, prior infection of nymphal ticks by either *A. phagocytophilum* or *B. burgdorferi* did not impact acquisition of either pathogen from the murine host suggesting little interaction between these pathogens in the tick vector once they are established in the tick. Interestingly, using a laboratory *Mus musculus* model of coinfection, Thomas et al. (2001) demonstrated that larval ticks fed on coinfected mice acquired both *B. burgdorferi* and *A. phagocytophilum* more effectively when compared to larvae that fed on single-infected mice. These contrasting observations could be due to differences in the immune responses of white-footed mice, the reservoir host, and laboratory mice to infections and co-infections. Ticks can feed on diverse mammalian hosts (McCoy et al., 2013), and it is critical to bear in mind that differential host immune responses may additionally impact infection prevalence in nature.

Highest prevalence of coinfections in ticks in nature are with *B. burgdorferi* and *Babesia microti* and is suggested to be in the range of 0-13% in nymphal ticks (Diuk-Wasser et al., 2016; Sanchez-Vicente et al., 2019). Hersh et al. (2014) suggest that this increased coinfection of *B. burgdorferi* and *B. microti* is aided in-part due to the reservoir competence of small mammalian hosts such as *Peromyscus leucopus* to be infected simultaneously with these two pathogens. Further, Dunn et al. (2014) showed that infection of *P. leucopus* with *B. burgdorferi* promotes infection, and transmission of *B. microti* from the mammalian host to the tick vector. Using a *Mus musculus* model and a different strain of *B. burgdorferi* Djockik et al. (Djokic et al., 2019) showed that *B. burgdorferi* restricts *B. microti* in coinfected mice and exacerbates Lyme disease. Despite the contrasting observations, these studies underscore potential interactions between the two pathogens in the mammalian host. Very little is understood of *B. microti*-*I. scapularis* interactions (Antunes et al., 2017). Given the increased prevalence of *B. burgdorferi*/*Babesia microti* coinfected ticks and the concurrent risk of human coinfections (Diuk-Wasser et al., 2016), it is important to determine how coinfections with *B. burgdorferi* and *B. microti* might influence the transmission of these pathogens to and from the tick. Although co-infections of *B. burgdorferi* with Powassan virus and *Borrelia miyamotoi* are less frequent (Tokarz et al., 2010; Diuk-Wasser et al., 2016), the gap in our understanding of the interactions of these pathogens in the tick vector and in the mammalian host needs to be addressed.

In addition to the interplay of different tick-borne pathogens, we must be aware that several genospecies of *B. burgdorferi* (*B. burgdorferi sensu lato* species) are maintained in nature enzootically (Swanson and Norris, 2008), including *B. burgdorferi* in the North America (Derdakova et al., 2004), and *B. burgdorferi*, *B. garinii* and *B. afzeli* in Eurasia (Misonne et al., 1998). There is also significant diversity within these genospecies (Rogovskyy and Bankhead, 2014) and this diversity is stably maintained in reservoir host populations ( (Swanson and Norris, 2008; Walter et al., 2016). Using two distinct *Borrelia* genospecies, *B. burgdorferi* and *B. afzelii*, Bhatia et al. (2018) showed that when infected nymphs feed on seropositive *Mus musculus*, the infectivity of the homologous spirochete strain is significantly attenuated within the tick gut facilitating the transmission of only the heterologous *Borrelia* strain to the animals. These observations highlight the seamless interaction between the host-vector and pathogen in the maintenance of *B. burgdorferi* diversity by ensuring the selection of rare variants of polymorphic surface antigens of the spirochete critical for spirochete fitness in natural hosts.

## Tick-*B. burgdorferi*-commensal microbiota interactions

The life cycle of *I. scapularis* offers ample opportunity for interaction with environmental microbiota that potentially include virus, protozoa, fungi and bacteria represented in soil and leaf litter (during their off-host phase) or on the skin of the mammalian host (during their on-host phase) (Narasimhan et al., 2021). de la Fuente et al. (2017) and Cabezas-Cruz et al. (2017) highlight the need to investigate the influence of non-pathogenic microbiota in ticks on the tick and *B burgdorferi*. Since the environment of the tick (be it lab colonies or field samples) is diverse, there is significant disparity among studies on the tick microbiome composition (Narasimhan et al., 2021). The emerging understanding is that there is no core tick microbiome, and that it is transient and variable (Ross et al., 2018). In addition to microbiota acquired from the environment, the tick also harbors endosymbionts that are transovarially transmitted and stably maintained in the tick (Kurtti et al., 2015). Since *I. scapularis* is an obligate hematophagous arthropod, its diet is lacking in essential B-vitamins including thiamine, pyridoxine, and folate (Duron et al., 2018) and the tick is likely dependent on its microbiota to provide these vitamins. It has been suggested that *Rickettsia buchneri*, the predominant endosymbiont of *I. scapularis*, provides the essential vitamins and is critical for tick fitness (Hunter et al., 2015). However, a recent study by Oliver et al. (2021) generated *R. buchneri*-free ticks by antibiotic treatment and showed that the absence of *R.buchneri* does not impact development or fecundity. It is likely that antibiotic treatment may not completely cure the ticks of *R. buchneri* in one cycle of antibiotic feeding. It is likely that a “stress” test might be more revealing of the role *R. buchneri* in tick biology. It is not known whether *R. buchneri* impacts *B. burgdorferi* acquisition ot transmission. Since *R. buchneri* is an intracellular bacterium and *B. burgdorferi* is an extracellular bacterium, a direct interaction is unlikely. An earlier study by Zhang et al. (2016) demonstrated that *B. burgdorferi* does not require thiamine or Vitamin B1, a key cofactor for most living organisms, for its growth and replication and that it may have evolved to live in a Vitamin B-constrained environment of the tick. Sonenshine and Stewart (2021) also raise the possibility that environmental bacteria that form the bulk of the ectosymbionts found in the midgut milieu are also capable of providing essential aminoacids, metabolites and vitamins to the tick and hence influence tick fitness and in-turn the vectorial capacity of the tick. Narasimhan et al. (2014) have shown that changes in the environmental microbiota composition influence larval engorgement and *B. burgdorferi* colonization of the tick. If so, changes in the composition of environmental microbiota in different regions of the USA would also influence infection prevalence in natural settings. Further, there is mounting evidence that the tick salivary transcriptome and proteome may be differentially expressed on different host species (Tirloni et al., 2017; Narasimhan et al., 2019). Whether this differential expression is signaled by host-specifc immune components in the bloodmeal or by the host skin microbiome and its metabolites at the skin-host interface remains to be investigated. This also underscores the impact of the host species on tick fitness and ultimately success of pathogen transmission. To fully understand how specific environmental microbiota manipulate the tick, approaches to generate aposymbiotic, and gnotobiotic ticks must be developed. Additionally, while tick microbiome studies have been largely bacteriocentric, it is important to examine other potentially key players including fungi, and viruses.

## Conclusions and future perspective

With the understanding that tick saliva contains several immunomodulators that facilitate tick feeding-a process essential for tick-borne pathogens to enter or exit the tick (Francischetti et al., 2009), the concept of saliva assisted transmission or SAT (Nuttall, 2019) has been at the centre stage of research on tick transmission of human pathogens. The availability of the genome sequence of *I. scapularis* (Gulia-Nuss et al., 2016) has allowed comprehensive cataloging of the salivary and midgut transcriptomes and proteomes and revealed new insights into tick biology and tick-host-pathogen interactions (Chmelar et al., 2016; de la Fuente et al., 2017; Wikel, 2018; Nuss et al., 2021). The last decade has brought into focus the role of the commensal and symbiotic microbiota associated with the tick in the context of tick-pathogen interactions (Bonnet et al., 2017; Narasimhan et al., 2021; Sonenshine and Stewart, 2021). Importantly, the geographic expansion of tick populations, potentially enhanced by climate change (Ogden et al., 2021), has signaled the need to understand how changes in abiotic factors influence the tick, and its pathogenic and commensal microbes. Thus, these multiples factors are entwined and together orchestrate pathogen movement to and from the mammalian host.

As we acknowledge that tick-pathogen interactions are orchestrated by biotic and abiotic factors, it also draws attention to the limitations of laboratory studies. Almost all data published on the influence of tick and *B. burgdorferi* gene expression have been performed with tick colonies raised under laboratory conditions. Ticks are routinely maintained at constant light cycling, humidity, and temperature conditions. The vast amount of knowledge gathered from controlled laboratory conditions indeed provide critical and foundational insights into tick-host-pathogen interactions. It is not feasible for laboratory studies to attempt to mimic field conditions that include diversity in host species and seasonal variations in abiotic factors and in biotic factors. Nevertheless, we must bear in mind that data collected from tick experiments performed in the laboratory may not fully recapitulate what ensues in ticks collected from the field. Future efforts may benefit from conducting “translational’ studies to calibrate and fine tune laboratory observations to address this limitation.

## Author contributions

JC and SN reviewed the literature, wrote sections of the manuscript. SS helped in collecting literature, and in writing. The complete manuscript was edited and revised by all co-authors. All authors contributed to the article and approved the submitted version.

## Funding

Funding was provided in part by the joint NIH-NSF-NIFA Ecology and Evolution of Infectious Disease award 1R01GM148992-01 and by the USDA National Institute of Food and Agriculture.

## Conflict of interest

The authors declare that the research was conducted in the absence of any commercial or financial relationships that could be construed as a potential conflict of interest.

## Publisher’s note

All claims expressed in this article are solely those of the authors and do not necessarily represent those of their affiliated organizations, or those of the publisher, the editors and the reviewers. Any product that may be evaluated in this article, or claim that may be made by its manufacturer, is not guaranteed or endorsed by the publisher.
